# Genome-Wide Identification and Gene Expression Analysis of Sweet Cherry Aquaporins (*Prunus avium* L.) under Abiotic Stresses

**DOI:** 10.3390/genes14040940

**Published:** 2023-04-19

**Authors:** Ariel Salvatierra, Patricio Mateluna, Guillermo Toro, Simón Solís, Paula Pimentel

**Affiliations:** Centro de Estudios Avanzados en Fruticultura (CEAF), Camino Las Parcelas 882, km 105 Ruta 5 Sur, Sector Los Choapinos, Rengo 2940000, Chile; asalvatierra@ceaf.cl (A.S.); pmateluna@ceaf.cl (P.M.); gtoro@ceaf.cl (G.T.); simon.solis.k@gmail.com (S.S.)

**Keywords:** aquaporin, major intrinsic protein, sweet cherry, *Prunus avium*, rootstock, abiotic stress

## Abstract

Aquaporins (AQPs) are integral transmembrane proteins well known as channels involved in the mobilization of water, small uncharged molecules and gases. In this work, the main objective was to carry out a comprehensive study of AQP encoding genes in *Prunus avium* (cv. Mazzard F12/1) on a genome-wide scale and describe their transcriptional behaviors in organs and in response to different abiotic stresses. A total of 28 non-redundant AQP genes were identified in *Prunus* spp. Genomes, which were phylogenetically grouped into five subfamilies (seven PIPs, eight NIPs, eight TIPs, three SIPs and two XIPs). Bioinformatic analyses revealed a high synteny and remarkable conservation of structural features among orthologs of different *Prunus* genomes. Several cis-acting regulatory elements (CREs) related to stress regulation were detected (ARE, WRE3, WUN, STRE, LTR, MBS, DRE, AT-rich and TC-rich). The above could be accounting for the expression variations associated with plant organs and, especially, each abiotic stress analyzed. Gene expressions of different PruavAQPs were shown to be preferentially associated with different stresses. PruavXIP2;1 and PruavXIP1;1 were up-regulated in roots at 6 h and 72 h of hypoxia, and in PruavXIP2;1 a slight induction of expression was also detected in leaves. Drought treatment strongly down-regulated PruavTIP4;1 but only in roots. Salt stress exhibited little or no variation in roots, except for PruavNIP4;1 and PruavNIP7;1, which showed remarkable gene repression and induction, respectively. Interestingly, PruavNIP4;1, the AQP most expressed in cherry roots subjected to cold temperatures, also showed this pattern in roots under high salinity. Similarly, PruavNIP4;2 consistently was up-regulated at 72 h of heat and drought treatments. From our evidence is possible to propose candidate genes for the development of molecular markers for selection processes in breeding programs for rootstocks and/or varieties of cherry.

## 1. Introduction

Aquaporins (AQPs) are integral transmembrane proteins that belong to the major intrinsic protein (MIP) superfamily. The first AQP was discovered in 1992 in humans, and called CHIP [1], and, since then, many members of this superfamily have been found in almost all living organisms. They are involved in transmembrane transport, facilitating the movement of water, small uncharged solutes and gases [2,3,4,5]. To date, five subfamilies had been identified in higher plants: plasma membrane intrinsic protein (PIP), tonoplast intrinsic protein (TIP), nodulin 26-like intrinsic protein (NIP), small basic intrinsic protein (SIP) and uncategorized X intrinsic protein (XIP) [3]. They assemble in tetramers, with each monomer acting as an independent channel [6].

Despite the large number of AQPs in nature, they all present a highly conserved “hourglass” shape and share some structural features such as six transmembrane helices, which are connected by five loops, named loop A, B, C, D and E. The NPA motif, named by asparagine, proline and alanine residues that are present in loop B and E inside the channel, assembles two half-helices, which constitute a seventh “pseudo-helix” transmembrane domain, creating an electrostatic repulsion of protons and a size barrier [7]. Another important conserved motif, called aromatic/arginine (ar/R), is a substrate selectivity filter, constituted of four residues and forming a narrow region inside the channel. Using statistical analysis of amino acid sequences of AQPs that transport glycerol, also called aquaglyceroporins (GLPs), five highly conserved residues were identified and determined as Froger’s positions (P1–P5) [8]. In addition, through a deep and comprehensive analysis of functionally characterized AQPs, nine amino acids called specificity-determining positions (SDPs) were identified for non-aqua substrates [9].

It has been shown that aquaporins can transport water and other uncharged solutes such as glycerol, urea, boric acid, silicic acid, hydrogen peroxide, ammonia, lactic acid and carbon dioxide [2,3,4,10]. Due to these important characteristics, aquaporins have been identified as key players in many processes in plants, among them cell elongation, pollen and seed germination, osmoregulation, stomatal and leaf movement, cytoplasmic homeostasis, and stress response [11,12,13,14,15]. Therefore, aquaporins are highly regulated during their development in a cell-specific manner by hormones and environmental signals [2]. Due to their relevance at the cellular and whole plant level, it is relevant to know the effect of different biotic stresses such as root hypoxia, salinity, high and lower temperatures expositions and drought on the aquaporin gene expression profiles to go ahead in the knowledge of how plants can adapt to adverse environmental conditions.

Climate change has affected agricultural production worldwide, with detrimental impacts on a huge number of crops. In the *Prunus* genus there are fruit species of commercial interest such cherry, peach, almond, plum and apricot. In commercial orchards of cherries, peaches and other stone fruits, scions of commercial varieties are grafted onto rootstocks, which can be obtained from the same or other *Prunus* species [16]. Rootstocks can confer tolerance to different abiotic stresses, improving the performance and yield of the scion [17,18].

Sweet cherry (*P. avium* L.) is an economically important crop that includes cherry trees cultivated for human consumption and wild cherry trees used for their wood, also called mazzards [19,20]. Its first available genome was published in 2017 by Shirasawa et al. [21], covering 77% of the total genome size, and generating new information for data mining, the construction of high-density linkage maps between sweet cherry and peach [22], genomic comparisons among Rosaceae species [23] and fruit ripening gene identification [24]. Recently, a high-quality genome assembly of *P. avium* L. was published [25], providing a good resource for further genetic and genomic studies. In this context, through a deep genome-wide analysis of *P. avium* L.; we present an approach that includes analyses of gene structure, sequence, amino acidic and conserved motifs, and gene expression profiles in different tissues and under several abiotic stresses on the cherry rootstock ‘Mazzard F12/1’, to deepen the understanding about the aquaporin gene family and its stress adaptive response in a woody fruit tree species, such as cherry.

## 2. Materials and Methods

### 2.1. Identification of Aquaporin Genes and Phylogenetic Analysis

The complete aquaporin family from *P. avium* L. was retrieved by data mining the genome assembly belonging to the ‘Tieton’ cultivar (Genbank accession GCA_014155035.1) available at NCBI, using the complete aquaporin family from *Prunus persica* as queries to perform local blastn to identify all the aquaporins. The aquaporins from *P. persica* were obtained by searching the major intrinsic protein domain “PF00230” (http://pfam.xfam.org/family/pf00230) in Phytozome V12.1. The aquaporins of *Prunus armeniaca*, *Prunus dulcis*, *Prunus salicina* and *Prunus mume* (GCA_903112645.1, GCA_902201215.1, GCA_000346735.1 and GCA_014155035.1 Genbank accession numbers, respectively) were also used for synteny analysis purposes. A phylogenetic tree was constructed using the deduced amino acid sequences as input in MEGA 11 software [26] using the maximum likelihood method with 1000 bootstrap.

### 2.2. Microsynteny Relationships of Four Prunus Species

A microsynteny map was built using the clicO FS tool (http://clicofs.codoncloud.com/) using orthologous genes of aquaporin from *P. avium*, *P. armeniaca*, *P. dulcis*, *P. mume*, *P. persica* and *P. salicina*.

### 2.3. Cis-Acting Regulatory Element Analysis

The 2000 bp genomic sequences upstream of the ATG of the aquaporin genes were downloaded and analyzed by PlantCARE (http://bioinformatics.psb.ugent.be/webtools/plantcare/html/) for cis-acting regulatory elements identification. The TBtools software was used for image visualization [27].

### 2.4. Gene Structure, Characteristic Motif, Physicochemical Features, In Silico Prediction of Transmembrane Domains and Subcellular Localization

The gene structure for each aquaporin was determined using the Gene Structure Display Server (http://gsds.gao-lab.org/). Multiple sequence alignment was performed using the MUSCLE tool [28] to determine the conserved residues of NPA, ar/R selectivity filter [29,30,31], Froger’s residues [8] and specificity-determining positions (SPD) [9]. Physicochemical characteristics were performed using ProtParam (http://web.expasy.org/protparam/). Prediction of subcellular localization was performed using Wolf PSORT [32] and Plant-mPLoc [33] with default parameters. The nonsynonymous (Ka) to synonymous (Ks) substitution rates were determined using the Ka/Ks tool (http://services.cbu.uib.no/tools/kaks). The grand average of hydropathy was predicted using the Protein GRAVY tool from the Sequence Manipulation Suite (http://www.bioinformatics.org/sms2/protein_gravy.html).

### 2.5. Abiotic Stress Treatments

One-year-old *P. avium* plants were used in the abiotic stress treatment. A set of 18 *P*. *avium* plants were individually placed inside of 1 L containers with a mix of peat moss:sand (2:1) as substrate and maintained in a growth chamber at 28 °C, 50% relative humidity, a photoperiod of 16/8 day/night and 150 μmol m^−2^ s^−1^ of incident photosynthetically photon flux density (PPFD). All the plants were maintained in these conditions during the treatments with an exception for cold and heat stress. Subsets of three plants were used for each treatment. For root hypoxia treatment, plastic containers were filled with water to approximately 4 cm above the pot substrate level, according to Pimentel et al., 2014 [34]. For salt treatment, plants were watered three times per week with a solution of 120 mM NaCl, according to Toro et al., 2021 [35]. For cold treatment, plants were exposed to 4 °C for 72 h. For heat treatment, plants were exposed to 40 °C for 72 h. Drought stress treatment was achieved by progressive substrate drying by applying irrigation with a volume of water equivalent to 90% of the weight of the substrate recorded the previous day until the early appearance of epinasty, which marked the reference point for the start of this treatment. For each abiotic treatment, roots and leaves were harvested at 6 and 72 h, immediately frozen in liquid nitrogen and stored at −80 °C for aquaporin gene expression.

### 2.6. RNA Extraction and Transcript Analysis by qRT-PCR

Three independent total RNA samples of individual plants were isolated for *PruavAQPs* expression analysis in different organs (roots, stems, leaves and flowers) and for expression analysis under abiotic stress treatments (roots and leaves) by using the CTAB method with minor modifications [36]. For total RNA extraction, 5 g of root or leaf tissue was used and the chloroform:isoamyl alcohol step was repeated three or more times depending of each sample. RNA quantity and quality were determined based on A260/280 and A260/230 wavelength ratios using an Infinite 200 PRO NanoQuant Spectrophotometer (Tecan, Männedorf, Switzerland). RNA integrity was confirmed on a 2% agarose gel and RedSafe staining. DNAse I (Ambion, Austin, TX, USA) was used to remove genomic DNA contamination. From each biological replicate, 2 mg of total RNA was used as a template for first-strand cDNA synthesis using the Maxima First Strand cDNA Synthesis kit (Thermo Scientific, Waltham, MA, USA). The cDNA was diluted 1:4, and 2 mL of the dilution was amplified by qRT-PCR, using isoform-specific primers designed with Primer Premier software 5.0 (Premier Biosoft International, Palo Alto, Santa Clara, CA, USA). The Mx3000 P qPCR System (Agilent Technologies, Santa Clara, CA, USA) and Maxima SYBR Green/ROX qPCR Master Mix (Thermo Scientific, Waltham, MA, USA) were used as per the manufacturers’ instructions. Each biological replicate was analyzed in duplicate. The specificity of the amplification products was confirmed by the registration of a single peak in melting curves. Each biological sample (three) contained two technical replicates. The *Prunus RNA polymerase II* (*Prupe.8 G132000.1*) was used to normalize the raw data and calculate relative expression levels [37]. The baseline (0 h) control treatment was used as the calibrator sample in the abiotic stress experiment. Normalized Ct values were used for determining gene expression, according to Pfaffl, 2001 [38]. The primers used in this study are listed in Table S1.

## 3. Results

### 3.1. Identification and Analysis of Aquaporin Genes in P. avium

In a previous study, the complete aquaporin family of *P. persica* were identified, with a total of 29 putative genes [39]. All those sequences were obtained using the first version of the *P. persica* genome, and most of them are incomplete. A search by PF00230 in Phytozome resulted in 29 genes in total, with full length sequences. The gene with code Prupe.3G009400, was discarded since it lacks one of the NPA motifs, resulting in 28 putative aquaporin genes in *P. persica*. These sequences were used as queries to search for aquaporins in the latest genome of *P. avium* available at NCBI, which resulted in 28 putative aquaporin genes. The same procedure was also used for identifying all aquaporins in *P. armeniaca*, *P. dulcis* and *P. mume* genomes, which resulted in the same 28 aquaporin genes in every species. These genes were unevenly distributed in almost all *P. avium* chromosomes (PAC). PAC1 contained three aquaporins, i.e., two NIPs (*PruavNIP4*;*1* and *PruavNIP7*;*1*) and one TIP (*PruavTIP4*;*1*), and PAC2 contained three PIPs (*PruavPIP1*;*1, PruavPIP1*;*2* and *PruavPIP2*;*1*) and two TIPs (*PruavTIP1*;*1* and *PruavTIP5*;*1*). PAC3 contained three NIPs (*PruavNIP2*;*1, PruavNIP4*;*2* and *PruavNIP6*;*1*), one TIP (*PruavTIP2*;*1*) and one SIP (*PruavSIP1*;*1*). PAC4 contained only *PruavNIP1*;*1*. PAC5 contained one member of NIP (*PruavNIP5*;*2*), one PIP (*PruavPIP1*;*3*) and two TIPs (*PruavTIP2*;*2* and *PruavTIP3*;*1*). PAC6 contained two members of the PIP subfamily (*PruavPIP2*;*2* and *PruavPIP2*;*3*) and one member of TIP (*PruavTIP1*;*2*). PAC7 contained one TIP (*PruavTIP1*;*3*), one NIP (*PruavNIP5*;*1*) and two SIPs (*PruavSIP1*;*2* and *PruavSIP2*;*1*). PAC8 contained one PIP (*PruavPIP2*;*4*) and two XIPs (*PruavXIP1*;*1* and *PruavXIP2*;*1*). As expected, these showed a high synteny with the other *Prunus* species, since most aquaporins were in the same chromosome number. The differences were found in the gene coding for NIP5;1 that was found on chromosome 3 in *P*. *armeniaca*, *P. persica* and *P*. *dulcis*. In addition, the gene coding for SIP1;1 was found on chromosome 1 in *P*. *dulcis*. The nomenclature used in our aquaporin names was based on the *Arabidopsis thaliana* aquaporin analysis [40], phylogenetic trees and chromosome location, maintaining a numerical order.

**Figure 1:**
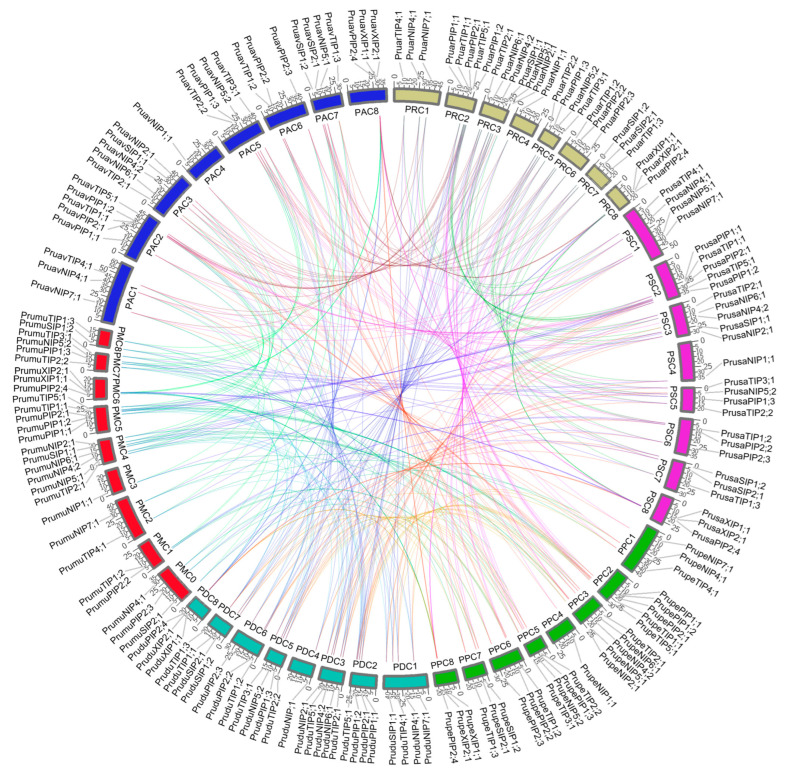
Analysis of synteny and chromosomal distribution of gene aquaporins from *Prunus* spp. PAC(1–8) represent the eight chromosomes from *P. avium*. The same case for *P. mume*, represented chromosomes as PMC(1–8), *P. persica* with PPC(1–8), *P. armeniaca* with PRC(1–8), *P. dulcis* with PDC(1–8) and *P. salicina* with PSC(1–8). Aquaporins with unplaced locations in *P. mume* are represented in PMC0 with reduced size so as to not distort the image.

Compared with *P. mume*, all aquaporins showed different positions, but with similar distribution in all subfamilies (Figure 1). In addition, comparing the number of identified aquaporins in *P. avium* with other plant species, it was inferior to *A*. *thaliana* with 35 aquaporins [40], *Zea mays* with 43 aquaporins [39], *Oryza sativa* with 34 putative aquaporins [41], *Fragaria vesca* with 39 putative aquaporins [39] and *Populus trichocarpa* with 58 putative aquaporins [42].

**Figure 2:**
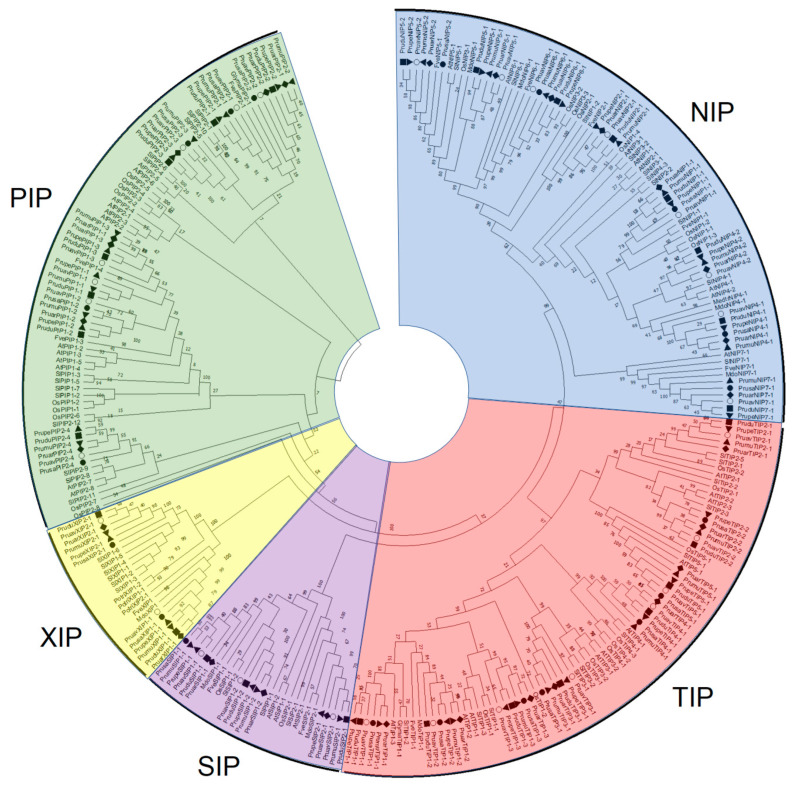
Phylogenetic tree of identified aquaporins in *P. avium* and other plant species. Open circles represent sequences from *P. avium*. Black dots represent sequences from *P. salicina*. Black squares represent *P. dulcis* sequences. Black rhombi represent sequences from *P. armeniaca*. Black triangles represent sequences from *P. mume*. Upside down black triangles represent sequences from *P. persica*.

Using the translated CDS of all genes, and using the peptide sequence of other plant aquaporins, a clustering algorithm was performed to determine the aquaporin subfamilies and the distribution of their members (Figure 2). The genome of *P. avium* contained members of five subfamilies of aquaporins identified in higher plants. In addition, while the NIP subfamily contained eight members, seven members were identified in the PIP subfamily, eight members in the TIP subfamily, three members in the SIP subfamily and only two XIP aquaporins (Figure 2).

*P*. *avium* aquaporins ranged from 236 to 314 amino acids in length, containing both classic NPA motifs, with a variation in the alanine position with S-T-L-V residues in NPA I and I-V in NPA II. PruavXIP1;1 was the only aquaporin with different NPA I and II motifs, consisting in SLV and SPA, respectively. In the case of the ar/R selectivity filter motif, the arginine residue was conserved in all members of the NIP, PIP and XIP subfamilies. Members from the SIP subfamily contained N-S residues and TIP members contained C-R-V amino acids instead. The Froger’s residues showed a higher conservation of amino acids for each subfamily; moreover, in the third position, the alanine was conserved in all aquaporins. On the other hand, almost all members were predicted to be localized at the plasma membrane, with 4 to 7 transmembrane domains and a molecular weight ranging from 25.08 to 33.85 kDa, isoelectric points from 4.78 to 9.75 and the Ka/Ks ratio less than 1.00 in all subfamilies. This latter would indicate that a stabilizing selection was maintaining the function of the aquaporin genes in *P*. *avium*. The GRAVY analysis showed positive values for all subfamilies, pointing out a hydrophobic nature of the aquaporins. Here, the members of the PIP subfamily averaged the lowest value, with 0.383, and the members of TIP subfamily with the highest value of 0.803, on average (Table 1).

### 3.2. Analysis of Cis-Acting Regulatory Elements of the Aquaporin Gene Family of P. avium

**Figure 3:**
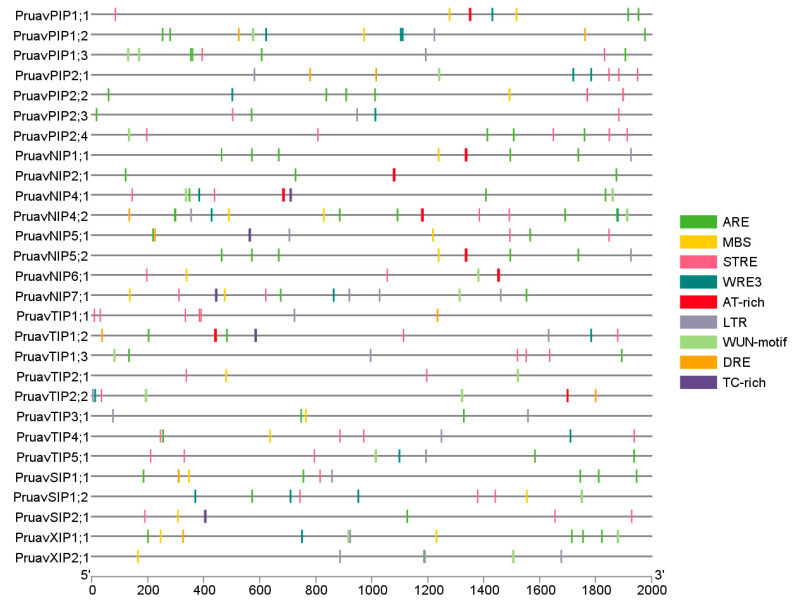
Analysis of cis-acting regulatory elements of the aquaporin gene family of *P. avium*. A total of 2000 bp promoter sequences of 28 aquaporin genes were analyzed using the PlantCARE database. Different color boxes represent the cis-acting regulatory elements for stress response.

The analysis of the 2000 bp upstream sequence of *P. avium* aquaporins identified several *cis*-acting regulatory elements (CREs) related to stress regulation (Figure 3). Twenty-three aquaporin genes contained CREs for anaerobic induction (ARE), where *PruavNIP6*;*1*, *PruavPIP2*;*1*, *PruavTIP1*;*1*, *PruavTIP2*;*1* and *PruavTIP2*;*2* were the exceptions. Twenty-one aquaporin genes contained at least one cis-acting element for wound (WRE3 and WUN), where *PruavNIP1*;*1*, *PruavNIP2*;*1*, *PruavNIP5*;*2*, *PruavSIP2*;*1*, *PruavTIP1*;*1*, *PruavTIP1*;*3* and *PruavTIP3*;*1* did not show any wound-related *cis*-acting element. Twenty aquaporin genes showed a general stress response (STRE), where *PruavNIP1*;*1*, *PruavNIP2*;*1*, *PruavNIP5*;*1*, *PruavNIP5*;*2*, *PruavPIP1*;*2*, *PruavTIP3*;*1*, *PruavXIP1*;*1* and *PruavXIP2*;*1* were exceptions. Nineteen aquaporin genes showed cis-acting elements related to low temperature responsiveness (LTR), but not in the case of *PruavNIP2*;*1*, *PruavNIP4*;*1*, *PruavNIP6*;*1*, *PruavPIP1*;*1*, *PruavPIP2*;*2*, *PruavPIP2*;*4*, *PruavSIP1*;*2*, *PruavSIP2*;*1* and *PruavTIP2*;*1*. Seventeen aquaporin genes contained a drought-inducible element (MBS), where *PruavNIP2*;*1*, *PruavNIP4*;*1*, *PruavPIP1*;*3*, *PruavPIP2*;*1*, *PruavPIP2*;*3*, *PruavPIP2*;*4*, *PruavTIP1*;*1*, *PruavTIP1*;*2*, *PruavTIP2*;*2* and *PruavTIP5*;*1* were the exceptions. Nine aquaporin genes, i.e., *PruavNIP4*;*2*, *PruavNIP5*;*1*, *PruavPIP1*;*2*, *PruavPIP2*;*1*, *PruavSIP1*;*1*, *PruavTIP1*;*1*, *PruavTIP1*;*2*, *PruavTIP2*;*2* and *PruavXIP1*;*1,* contained the *cis*-acting element for dehydration (DRE). Nine aquaporin genes contained elicitor-mediated activation elements (AT-rich), i.e., *PruavNIP1*;*1*, *PruavNIP2*;*1*, *PruavNIP4*;*1*, *PruavNIP4*;*2*, *PruavNIP5*;*2*, *PruavNIP6*;*1*, *PruavPIP1*;*1*, *PruavTIP1*;*2* and *PruavTIP2*;*2*. Finally, only five aquaporin genes contained a cis-acting element involved in defense and stress responsiveness (TC-rich), i.e., *PruavNIP4*;*1*, *PruavNIP5*;*1*, *PruavNIP7*;*1*, *PruavSIP2*;*1* and *PruavTIP1*;*2*.

### 3.3. Analysis of Exon–Intron Structure

**Figure 4:**
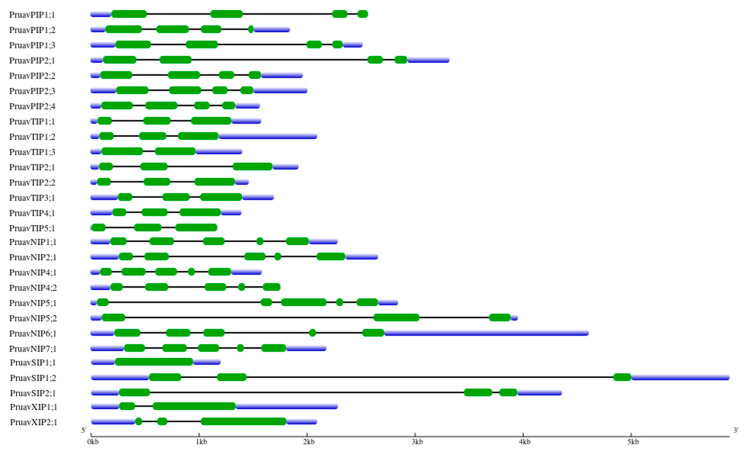
Exon–intron structure analysis of 28 *P. avium* aquaporins. Green rectangles represent exons. Blue rectangles represent the 5′ and 3′ UTR. Lines represent introns.

The 28 identified aquaporin genes were also analyzed using a constructed gene model (Figure 4). The shortest exon was found in *PruavPIP1*;*2*, with 33 bp, and the longest one was found in *PruavXIP2*;*1*, with 792 bp. The shortest intron was found in *PruavNIP4*;*1*, with 91 bp, and the longest intron was found in *PruavSIP1*;*2*, with 3450 bp. The number of introns varied from none in *SIP1*;*1* to a maximum of four in members of the *NIP* subfamily (Figure 4).

The structures of the aquaporin genes from the NIP subfamily were conserved in almost all members, containing four introns and five exons, with the exception observed in the *PruavNIP5*;*2* gene, which showed two exons. In the PIP subfamily*,* all members showed the same number of introns and exons, denoting a high conservation of gene structure. In the SIP subfamily, *PruavSIP1*;*2* and *PruavSIP2*;*1* contained two introns and three exons, and the lengths of the exons were similar. It is noteworthy that the third member, *PruavSIP1*;*1,* evidenced an absence of introns. In the TIP subfamily, it was observed that all genes contained two or three exons, and their lengths were similar. The exception was *PruavTIP1*;*3* which contained only one intron and two exons. Finally, introns and exons of the members of the XIP subfamily were different between them regarding their numbers and lengths; thus, PruavXIP1;1 contained one intron and two exons, while PruavXIP2;1 contained two introns and three exons (Figure 4).

### 3.4. Structural Characteristics of P. avium Aquaporins

#### 3.4.1. *P. avium* NIP Subfamily

The members from this subfamily had a protein sequence length of 244 to 307 residues, and an identity ranging from 33.80 to 64.1%. The two NPA motifs presented slight variations in the third amino acid, changing the well-known alanine to serine in PruavNIP5;1, PruavNIP5;2 and PruavNIP6;1 at the first NPA (Table 1). In the case of the second NPA, PruavNIP5;1 had an isoleucine in the third amino acid, while PruavNIP5;2 and PruavNIP6;1 both had a valine. The ar/R motifs also were less conserved. They contained [W/G/S/T/A]-[V/S/I]-[A/G]- R residues as selectivity filters, except for PruavNIP5;2, which lacked the second ar/R residue. The subcellular localization was predicted to be similar for all members, where the Wolf PSORT tool predicted a localization at the plasma membrane, except for PruavNIP5;2, whose location was predicted at the vacuole level. Meanwhile, Plant mPloc predicted the localization in the cell membrane for all members of this subfamily. With an average isoelectric point and molecular weight of 8.06 and 29.54, respectively, almost all members belonging to this group had a prediction of six transmembrane domains, except for PruavNIP5;2, with five predicted domains. Regarding the Froger’s residues, the five residues contained little variation within this subfamily, with phenylalanine or leucine in position 1 (P1), except for PruavNIP7;1, with a tyrosine in this position. P2 contained serine or threonine, while P3 and P4 contained alanine and tyrosine in all members. P5 was the least conserved, with the presence of isoleucine, leucine, valine and phenylalanine residues (Table 2).

Based on SDP analysis, all NIPs were categorized as boric acid transporters, but PruavNIP1;1 had a substitution of M for I/L/T in SDP7, and PruavNIP7;1 had a substitution of I for T/V in the SDP1. In the H_2_O_2_-type SDPs, four members of NIPs were categorized as H_2_O_2_ transporters, i.e., PruavNIP2;1, PruavNIP4;1, PruavNIP5;1 and PruavNIP5;2. Only one aquaporin from the NIP subfamily was inferred to be a silicic acid transporter (PruavNIP2;1), with a substitution of V for A/E/L in SDP3 and a substitution of N for A/K/P/T in the SDP9 position. Finally, only four members of the NIP aquaporins, i.e., PruavNIP1;1, PruavNIP2;1, PruavNIP5;1 and Pruav5;2, were identified as putative urea transporters (Table 2).

#### 3.4.2. *P. avium* PIP Subfamily

The PIP subfamily showed a canonical signature of water channel motifs. The sequence identity ranged from 68.25% to 93.01%, with an amino acid sequence length between 281 and 290 residues. PIP aquaporins showed highly conserved dual NPA motifs with no variation among the members. The selectivity filter (ar/R motif) showed a characteristic configuration of water-channel proteins (FHTR), and this motif was conserved in all members of this subfamily. With an average isoelectric point and molecular weight of 8.65 and 31.15, respectively, all members of this subfamily were predicted to be localized in the plasma or cell membrane, with six transmembrane domains predicted, except for PruavPIP1;1, with only five. The Froger’s residues showed conserved amino acids in P2–P5 positions (SAFW), but PruavPIP1;2 was the only with a glutamine residue instead of the glutamic acid at P1 (Table 1).

As boric acid transporters, all PIPs were categorized as functional transporters, with a slight difference between PIP1 and PIP2 at SDP6. PIP1s have an L and PIP2s have an I at this specific position. Interestingly, in the CO_2_-type SDPs, only PruavPIP1;1, PruavPIP1;3 and PruavPIP2;2 were inferred to be transporters of CO_2_ with different residues at the SDP3 position, whereas PruavPIP1;1 and PruavPIP1;3 evidenced a substitution of T for C and PruavPIP2;2 had a substitution of S for C. Finally, all PIPs were categorized as H_2_O_2_ transporters and urea-type SDPs (Table 2).

#### 3.4.3. *P. avium* TIP Subfamily

As seen for the NIP subfamily, the TIP subfamily showed the highest number of members with eight aquaporins. The sequence identity varied from 42.91% to 81.71%, and sequence length ranged between 248 and 256 amino acids. NPA motifs did not present variations. The ar/R filter showed differences among the members, with H, I, A, V residues for PruavTIP1 proteins. PruavTIP2 proteins showed H, I, G, R residues. PruavTIP3;1 and PruavTIP4;1 showed the same residues of TIP1 proteins but changing the fourth amino acid by R. PruavTIP5;1 showed a different ar/R motif with N, V, G, C residues in its selectivity filter. In the transmembrane domain prediction, most TIPs contained six domains, with PruavTIP2;1 and PruavTIP4;1 with seven predicted domains. On the other hand, most TIP aquaporins were localized in the plasma membrane and vacuole, with an average molecular weight of 25.98 and an isoelectric point ranging between 4.78 and 7.11. The Froger’s residues were highly conserved in most TIP aquaporins, with the residues T, S, A, Y, W in P1–P5 positions. However, PruavTIP3;1 varied in the P2 residue, changing the S for A. In addition, PruavTIP5;1 showed different residues at the P1 and P2 positions, containing V and A instead of T and S (Table 1).

Based on SDP analysis, only PruavTIP2;1 was inferred to be an ammonia transporter, differing in three amino acids in the SDP3, SDP6 and SDP9 positions, with T-L-I-L-A-T-H-P-V for all residues. Almost all members of the TIP subfamily (with the exception of PruavTIP1;2 and PruavTIP4;1) were categorized as hydrogen peroxide transporters, with PruavTIP2;1 and PruavTIP2;2 having a different residue in SDP6, containing an N in this position, and PruavTIP5;1 containing a T in SDP5. Finally, all TIPs were categorized as urea-type SDPs (excluding PruavTIP1;2) (Table 2).

#### 3.4.4. *P. avium* SIP Subfamily

Three aquaporins were identified for this subfamily. Their sequence lengths varied from 236 to 244 aa and their identities ranged between 31.88% and 60.67%. Only the second NPA motif was conserved among those proteins. In detail, PruavSIP1;1 had a serine instead of an alanine in the third residue and PruavSIP1;2 and PruavSIP2;1 had a threonine and a leucine, respectively. The selectivity filter ar/R was more conserved in both SIP1s with A/T-V-P-N, while PruavSIP2;1 had S, H, G, S amino acids in this case. In addition, Wolf PSORT predicted the subcellular localization in the vacuole, chloroplast and plasma membrane, while Plant mPloc predicted a cell membrane for all members and a vacuole for SIP1 aquaporins. Despite the differences evidenced by these transmembrane domain predictions, SIP aquaporins shared similar isoelectric points and molecular weights. The Froger’s residues were conserved in SIP1 with M, A, A, Y, W residues, while only SIP2 contained F, V, A, Y, W amino acids. Based in the SDPs analysis, the members of this subfamily did not show any important residues to be determined as a potential transporter for other molecules different from water.

#### 3.4.5. *P. avium* XIP Subfamily

The only two members from this subfamily showed sequence lengths between 304 and 314 aa. PruavXIP1;1 shared a 40.6% sequence identity with PruavXIP2;1. The NPA motifs were highly variable. Thus, the first NPA changed to SLV and NPV in PruavXIP1;1 and PruavXIP2;1, respectively. The second NPA conformed with an SPA in PruavXIP1;1 and the canonical NPA in PruavXIP2;1. The ar/R filter showed differences between both XIPs, since PruavXIP1;1 showed V, I, V, R residues and PruavXIP2;1 showed differences at the first and second positions (I, T, V, R). Both proteins were predicted to be localized in the plasma or cell membrane. The number of predicted transmembrane domains was six for PruavXIP1;1 and seven for PruavXIP2;1. Their molecular weight and isoelectric point averaged 33 and 6.86, respectively. The Froger’s residues were highly conserved, with only a point variation in P1, with [M/V]-C-A-F-W residues in this subfamily (Table 1).

Both XIP aquaporins were also categorized as boric acid transporters, but only PruavXIP1;1 showed different residues, given by a substitution of L for I/V in SDP2 and a substitution of V for I/L in SDP6. SDP analysis suggested that only PruavXIP2;1 can transport H_2_O_2_. PruavXIP1;1 represented a novel urea-type SDP with the substitution of V for L/M in SDP5 and substitution of G for A/G/P in SDP6 (Table 2).

### 3.5. Expression Pattern of PruavAQP Genes in Different Organs

**Figure 5:**
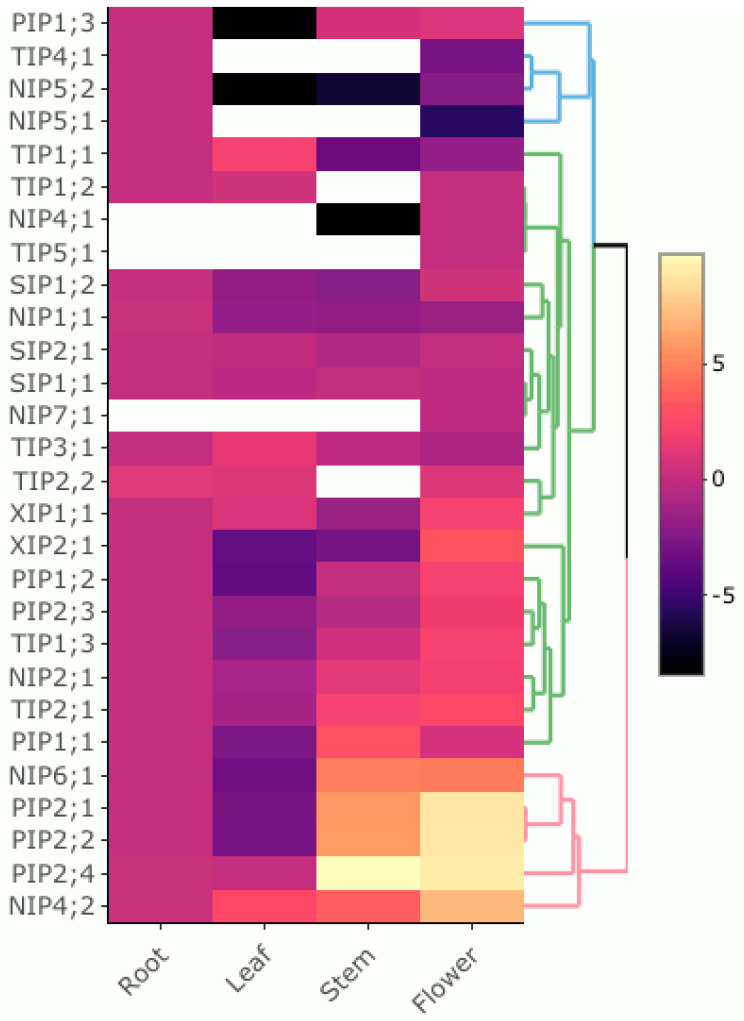
Heatmap and hierarchical clustering of the expression profiles of all AQP genes in different cherry tissues. The expression abundance of each transcript (log2 of qPCR analysis fold-change values, three biological and two technical replicates) is represented by a color range from yellow (higher expression) to black (lower expression). White: not detected at transcript levels. For each AQP gene, transcript levels were calculated using its mRNA abundance detected in roots as reference for the rest of the organs.

Roots, leaves, stems and flowers were used for expression patterns of 28 PruavAQPs by qRT-PCR (Figure 4). All Pruav aquaporins were detected in at least one organ. Flowers and roots were the organs with the largest number of expressed aquaporins, with 28 and 25 aquaporin genes, respectively. On the other hand, the lowest number of aquaporins detected was in stems. Some aquaporins showed a highly organ-associated expression. Among *NIP* genes, *PruavNIP1*;*1*, *PruavNIP5*;*1* and *PruavNIP5*;*2* were preferentially expressed at root level and the expressions of *PruavNIP4*;*1* and *PruavNIP7*;*1* were exclusively detected in flowers. Of all NIPs, only *PruavNIP4*;*2* was up-regulated in leaves. *PruavNIP4*;*2* and *PruavNIP6*;*1* were strongly expressed in stems and flowers (Figure 5).

All *PruavPIPs* were expressed in roots. *PruavPIP1*;*1*, *PruavPIP2*;*1*, *PruavPIP2*;*2* and *PruavPIP2*;*4* were strongly up-regulated in stems. Several *PruavPIPs* were down-regulated in leaves. In addition, all *PruavPIPs* were expressed in flowers, with *PruavPIP2*;*1*, *PruavPIP2*;*2* and *PruavPIP2*;*4* being strongly expressed in this organ (Figure 5).

Almost all *PruavTIPs* were expressed in roots, excluding *PruavTIP5*;*1,* which was only expressed in flowers. Only *PruavTIP1*;*3* and *PruavTIP2*;*1* were up-regulated in stems while *PruavTIP1*;*1* and *PruavTIP3*;*1* were down-regulated. On the other hand, *PruavTIP1*;*2*, *PruavTIP2*;*2*, *PruavTIP4*;*1* and *PruavTIP5*;*1* were not detected in stems. *PruavTIP1*;*1* and *PruavTIP3*;*1* were strongly expressed in leaves and *PruavTIP1*;*3* and *PruavTIP2*;*1* in flowers (Figure 5).

All *PruavSIPs* were expressed in roots. *PruavSIP1*;*1* was expressed in stems but *PruavSIP1*;*2* and *PruavSIP2*;*1* were down-regulated. *PruavSIP1*;*1* and *PruavSIP1*;*2* were repressed in leaves and only *PruavSIP1*;*1* was down-regulated in flowers (Figure 5). Noteworthy, the expression profile of both *PruavXIPs* was quite similar. *PruavXIP1*;*1* and *PruavXIP2*;*1* were expressed in roots, strongly up-regulated in flowers and repressed in stems. However, an opposite pattern was detected in leaves since *PruavXIP1*;*1* was up-regulated and *PruavXIP2*;*1* was repressed (Figure 5). Finally, it should be noted that *PruavPIP1*;*3*, *PruavTIP4*;*1*, *PruavNIP5*;*1* and *PruavNIP5*;*2* showed very similar transcriptional profiles, which were characterized by their expression associated preferentially with the root, together with little or no presence of transcripts in the other organs assessed. Likewise, *PruavNIP6*;*1*, *PruavPIP2*;*1*, *PruavPIP2*;*2*, *PruavPIP2*;*4* and *PruavNIP4*;*2* were grouped into a clade defined by their marked gene expression in stems, being even higher at the flower level (Figure 5).

### 3.6. Expression Profiles of AQPs Genes in Response to Different Abiotic Stresses

**Figure 6:**
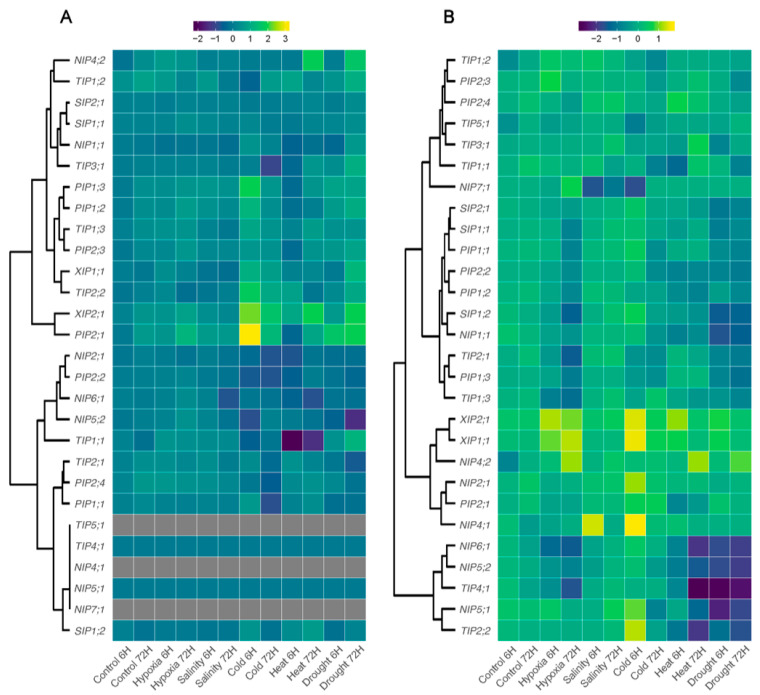
Heatmap and hierarchical clustering of the expression profiles of all AQP genes under different abiotic stress treatments. The expression abundance of each transcript (log2 of qPCR analysis fold-change values, three biological and two technical replicates) is represented by a color range from yellow (higher expression) to dark blue (lower expression). Gray: not detected transcript levels. For each AQP gene, transcript levels were calculated using its mRNA abundance detected in the control condition (6 h) as reference for the rest of the samples. (**A**) leaf, (**B**) root.

We analyzed the expression patterns of *PruavAQP* genes in roots and leaves under different abiotic stresses: hypoxia, salinity, cold, heat and drought. We evaluated two sampling times (6 and 72 h) to find out if there were aquaporins related to early or late responses. When comparing the expression profiles of all *AQP* genes under different stresses analyzed, we observed that there were more *PruavAQP* genes that were down-regulated in roots. Interestingly, three *PruavAQP* were not detected in leaves (*PruavTIP5*;*1*, *PruavNIP4*;*1*, *and PruavNIP7*;*1*) independently of the abiotic stress analyzed (Figure 6).

In roots under cold and salt stress, 50% and a 42.9% of *PruavAQP* genes were up-regulated, respectively. On the other hand, in roots under drought stress, more than 60% of all AQP genes at 6 and 72 h were down-regulated. In leaves, 65.2% were up-regulated under hypoxia stress at 6 h, and 60.9% of AQP genes were up-regulated under drought stress at 72 h. In addition, 47.8% of *PruavAQP* genes were early down-regulated in leaves under heat stress and a 47.8% were repressed in salinity stress 72 h later (Figure 6).

In roots exposed to hypoxia stress, a large number of genes (19) were down-regulated (all *PruavPIPs*, five *PruavNIPs*, four *PruavTIPs* and all *PruavSIPs*). Only five aquaporin genes were up-regulated under hypoxia stress at 6 and 72 h, viz.; *PruavNIP4*;*2*, *PruavNIP7*;*1*, *PruavTIP1*;*2, PruavXIP1*;*1* and *PruavXIP2*;*1*. However, the expression pattern in leaves was quite different since 13 genes were up-regulated and only 7 genes down-regulated (Figure 6).

Salinity stress modified the expression profile of aquaporin genes in roots, evidencing 17 aquaporin genes as up-regulated and only 7 as down-regulated (*PruavNIP1*;*1*, *PruavNIP5*;*1*, *PruavNIP5*;*2*, *PruavNIP6*;*1*, *PruavTIP1*;*1*, *PruavTIP4*;*1* and *PruavSIP1*;*2*). Among them, only *PruavTIP1*;*1* was down-regulated at 72 h of salt stress and the rest of the aquaporin genes were down-regulated earlier (6 h). In leaves, a similar number of *PruavAQPs* were down- and up-regulated in response to salinity (Figure 6).

Cold stress also altered the expression pattern of *PruavAQPs* in roots: 14 genes were up-regulated at 6 h and 15 genes were down-regulated at 72 h of treatment. In leaves, a similar number of aquaporins were up- and down-regulated, independent of the analyzed sampling time (Figure 6).

Notably, two stresses, heat and drought, showed similar expression profiles in roots. In this organ, most of the *PruavAQPs* were down-regulated under heat stress at 6 and 72 h. A few genes were up-regulated in roots under these extreme conditions: *PruavNIP4*;*2*, *PruavPIP1*;*3*, *PruavPIP2*;*3*, *PruavPIP2*;*4* and *PruavTIP3*;*1*. In drought, most of the root aquaporins were repressed and only *PruavNIP4*;*2*, *PruavPIP2*;*1*, *PruavXIP1*;*1* and *PruavXIP2*;*1* were up-regulated. In leaves, *PruavAQPs* genes were mainly down-regulated under heat stress. However, under drought stress, several *PruavAQP* genes evidenced an up-regulated gene expression at 72 h (Figure 6).

## 4. Discussion

According to the IPCC Sixth Assessment Report (AR6), in the most recent five decades, the global surface temperature has experienced the most rapid increment estimated for any 50-year period over the last 2000 years, and is a phenomenon widely related to emissions of greenhouse gases derived from human activities [43]. An econometric analysis that evaluated the impacts of different climate scenarios on global agricultural productivity concluded that world agricultural productivity has become vulnerable to climate change, resulting in a global productive decline that averages 21% in the last 60 years [11]. The ability of crops to adapt to adverse environmental conditions is a key factor in sustaining food production. Plants deploy a wide variety of molecular and physiological mechanisms to ensure the acquisition of resources and their most efficient use in response to stressful stimuli, such as those imposed by the challenges of climate change.

As transmembrane channels involved in the selective bidirectional mobilization of water, ions and uncharged low-molecular mass solutes [2,3,4,10] through osmotic gradients, aquaporins (AQPs) have been shown to play a crucial role in the uptake of water and nutrients from the environment and their distribution throughout the whole plant.

Due to their function of transporting resources, aquaporins are ubiquitous protein channels that participate in various developmental and stress response processes [11,12,13,14,15,44,45]. Thus, the regulation of gene expression of the numerous isoforms of aquaporins in response to stresses such as root hypoxia [10], drought [46,47], salinity [48,49], cold [50,51,52,53], heat [54,55] and combinations of them [56] have been consistently reported.

### 4.1. Identification and In Silico Characterization of the PruavAQP Gene Family

The rapidly growing number of genome sequencing initiatives has allowed the research of many important economic plants in a deeper manner [57,58,59,60,61,62,63,64]. To date, seven genomes of species from the *Prunus* genus are publicly available, i.e., *P. avium*, *P. armeniaca*, *P. dulcis*, *P. mume*, *P. persica*, *P.s salicina* and *P. yedoensis*. The latter was excluded from the synteny analysis since it is reported as contigs, impeding the comparative study with the other *Prunus* species at the chromosomal level. A genome-wide identification of gene aquaporins in different plant species, including *P. persica*, showed a total of 29 putative aquaporins in the first genome assembly [39]. However, in the later and more curated version 2.1, available in Phytozome v12.1, we identified 28 sequences as putative aquaporins. Chen et al., 2018 [65] reported 25 putative full-length aquaporin genes in a first approach to identify and analyze the aquaporin family in *P. avium*. However, our methodology identified 28 full-length putative aquaporins genes, the same number as reported for other *Prunus* species, with a high sequence identity among the six species compared in this study. This denotes a high conservation of the aquaporin family within the *Prunus* genus. In addition, the microsynteny analysis showed a high synteny among *P. avium*, *P. armeniaca*, *P. dulcis*, *P. persica* and *P. salicina*, but with a similar locus pattern of distribution for almost all gene aquaporins in *P. mume* (Figure 1). The clustering algorithm presented in the phylogenetic tree allowed us to classify our aquaporins clearly within the five subfamilies and their subgroups described for higher plants (Figure 2).

Since aquaporin proteins can play a crucial role in water and other solute transport across plant membranes, their expression can be modulated by various abiotic and biotic stresses [66]. The identification of cis-acting elements related to stress regulation in the upstream sequences of *PruavAQP* genes revealed that, excluding *PruavPIP2*;*1*, *PruavTIP1*;*1*, *PruavTIP2*;*1*, *PruavTIP2*;*2* and *PruavNIP6*;*1*, almost all aquaporins contained the ARE motif, denoting susceptibility to be induced by anaerobiosis. This pattern is like what was found in *Cucumis sativus*, where members of the same subfamily (i.e., *CsPIP1*;*1*, *CsPIP2*;*1*, *CsTIP4*;*1*, and *CsNIP3*;*1*) lacked the ARE motif [67]. However, a different pattern was observed in *Lycium barbarum*, where 16 out of 38 of their aquaporins did not contain the ARE motif [55]. Some aquaporin genes were exceptions to other stress-responsive cis-acting elements. Overall, these findings suggest that different stresses can activate different sets of cis-acting elements in sweet cherry aquaporins, leading to differential expression of these genes and, consequently, modulating the transport of water and other solutes in response to specific stress conditions (Figure 3).

On the other hand, when comparing the gene structure of *P. avium* aquaporins (Figure 4) with other species, we noted that there was a high conservation in the number of introns for each aquaporin subfamily with *Solanum lycopersicum* [57], *Glycine max* [68], *Hebea brasiliensis* [69], *Phaseolus vulgaris* [70], *Citrus sinensis* [71], *Musa acuminata* [72] and *Sorghum bicolor* [73].

One of the first important motifs described was the NPA, which is highly conserved among the aquaporins in *P. avium*, although with some variations (Table 1). Thus, the PIP and TIP subfamilies were the only ones with the three residues (asparagine, proline and alanine) present in all their members. In addition, not only the NPA motif was conserved in the PIP subfamily, but also the residues that form the ar/R selectivity filter. Compared with other species, those amino acids were also fully conserved in *Z. mays*, *A. thaliana*, *P. trichocarpa*, *S. lycopersicum*, *G. max* and *B. rapa* [42,57,68,74,75,76]. Previous studies have suggested its importance in the function of this subfamily, which could be regulating water uptake in roots and leaves [11,77], CO_2_ transport in the mesophyll in *N. tabacum*, *A. thaliana*, *Hordeum vulgare* and *O. sativa* [44,78,79,80], and even enhancing drought and salt tolerance in transgenic *A. thaliana* [81]. The Froger’s residues (Table 1) were also consistent with those proposed for water channels, with PruavPIP1;2 and PruavPIP1;3 with a variation at P1 (glutamic acid instead of glutamine), which could be affecting the channel conformation and, consequently, the transport performance due to the electric charge of the glutamic acid. The SDP analysis (Table 2) also inferred that PIPs were transporters for solutes. PIP aquaporins from *O*. *sativa* have been experimentally tested as boron transporters [82,83]. Here, all PruavPIPs were predicted as boric acid transporters, with no amino acid variations from those proposed for such a function. As CO_2_ transporters, some sequence variations were detected in SDP3, with a threonine in the case of PruavPIP1;1 and PruavPIP1;3, and a serine in PruavPIP2;2 instead of a cysteine. The shift of a non-polar by a polar residue could imply an altered efficiency for water transport given by a different channel conformation due to polar groups having more molecular freedom under aqueous conditions. Members of PIP1 and PIP2 subgroups have been characterized as H_2_O_2_ and urea transporters in *A*. *thaliana* and *Z*. *mays* [84,85], respectively. In our SDP analysis, all PIP members were predicted as potential transporters for those molecules, containing all the residues proposed with no variation. Previous studies on spinach aquaporin SoPIP2;1 and *A*. *thaliana* AtPIP2;4 characterized them as water and hydrogen peroxide transporters [86,87]. Interestingly, a gating mechanism mediated by a conformational change of the cytoplasmatic loop D was evidenced on SoPIP2;1, which consisted in a very conserved motif (S-A-T-D-[P,A]-K-R-[N,S]-A-R-D-S-H-V-P-[I,V]-L-A-P); such residues were present in both Pruav PIP aquaporin subfamilies. Moreover, the GRAVY analysis (Table 1) also showed the best value for hydrophilic affinity and, considering the low Ka/Ks ratio in this subfamily, a potential water transport capacity was expected for all PruavPIP aquaporins.

The NIP subfamily can be divided into three functional subgroups (NIP I-III), which are based in the four residues that form part of the ar/R selectivity filter (Wallace and Roberts, 2004) [88]. NIPs have a wide range of substrate specificity such as water, arsenic, silicic acid, ammonia, glycerol, urea, boric acid, silicon, aluminum and lactic acid [10,52,89,90,91,92,93,94,95,96,97,98]. In our Pruav NIP sequences analysis, we evidenced that this subfamily had the most variation in the ar/R selectivity filter and Froger’s residues. Moreover, three members (PruavNIP5;1, PruavNIP5;2 and PruavNIP6;1) exhibited a substitution in the third amino acid in both NPA motifs. However, the SDP analysis revealed that the whole PruavNIP subfamily was inferred to be a boric acid transporter, with a high residue conservation in all SDPs, suggesting a similar function for all NIPs. The *A*. *thaliana* boric acid transporter AtNIP5;1 spans NPS/NPV motifs and A/I/G/R residues in the ar/R selectivity filter with low water permeability [85]. In our study, its ortholog PruavNIP5;2 shared the same residues in the NPAs motifs, which suggests a function as a boric acid transporter in *P. avium*, although the second residue in the Ar/R filter was lacking. Another boron transporter in Arabidopsis is AtNIP6;1, with no water permeability [96]. Its ortholog PruavNIP6;1 showed a higher GRAVY value (i.e., more hydrophobic) than PruavNIP5;2 but there was a threonine instead of alanine in the H2 position of the ar/R selectivity filter, which could indicate a different transport efficiency due to a less wide pore produced by steric hindrance of the threonine size. OsNIP2;1 has been reported as a silicon, arsenite and boron transporter, with double NPA and G/S/G/R selectivity filter motifs [93]. The *P. avium* homolog, PruavNIP2;1, shared the same residues as well as the nine SDPs, which also suggests its involvement in boron transport. The NIP subgroup I (NIP I) can transport water, glycerol, lactic acid, ammonia, arsenite and hydrogen peroxide. PruavNIP I channels contained W V/I A R residues in the ar/R selectivity filter. In a previous work, our research group characterized PruavNIP1;1 as a lactic acid transporter [10], spanning WVAR amino acids just like PruavNIP4;1 and PruavNIP4;2. In this case, their orthologs AtNIP4;1 and AtNIP4;2 played a key role in the pollen development and pollination, transporting not only water but also ammonia, urea, boric acid and hydrogen peroxide in the case of AtNIP4;1 [83]. They shared the same NPA and ar/R motifs, but a different Froger’s residue at P1, with a phenylalanine in both AtNIP4s instead of leucine, as in the case of their homologs of *P. avium*.

In the TIP subfamily, TIP1 members have lost the arginine from the LE2 position, replacing it with a valine. This could affect the constriction zone with a wider pore due to the difference of size between these amino acids, allowing the pass for bigger non-aqueous molecules and affecting the transport rate of the channel. The same can be said for PruavTIP5;1, which has also lost the arginine, replaced by a smaller cysteine residue. Its ortholog, AtTIP5;1, has been characterized as a water and urea transporter [99,100]. Both share the same amino acids for NPA, ar/R selectivity filter, Froger and SDP residues for urea transport, suggesting a similar function for PruavTIP5;1. Previous studies have analyzed the importance of the arginine in the LE2 position, where its mutation altered the transport capacity of TIPs in *Tulipa gesneriana* [101], but mimicking the ar/R selectivity filter of the functional characterized TIP ammonia transporter from Arabidopsis PIP aquaporins resulted in no evidence of ammonia transport [102]. This suggests that the ar/R selectivity filter is not the most important region of the channel. Aside from the NPA, ar/R, Froger and SDP residues, it is important to consider the residues that form part of the external sides of the channels [5]. In our previous study, we analyzed the electrostatic surface potential in the 3D model of the lactic acid transporter PruavNIP1;1, which is similar to AtNIP2;1 [10], empirically reported as a lactic acid transporter [95]. This is a relevant feature since it can predict what kind of molecules will have better affinity with the cytosolic residues before it enters the channel.

Unlike any other aquaporin subfamily in higher plants, the SIP aquaporins subfamily have not been widely studied in term of structure and function. Just like *A*. *thaliana*, *P. avium* contained three genes encoding for SIP aquaporins: two SIP1 and one SIP2. Members of this subfamily in *A*. *thaliana* have been localized in the endoplasmic reticulum, and the ability of SIP1s for water mobilization has been assessed in Arabidopsis [103] and *Vitis vinifera* SIPs [104]. Molecular dynamic simulations in the 3D model of PruavSIP1;2 showed water transport across the channel (Mateluna, in preparation), and only differed from PruavSIP1;1 in the third residue of the first NPA, containing a threonine and serine, respectively. These residues are polar and can form interactions with water molecules, suggesting a similar function for both aquaporins. PruavSIP2;1 contained a leucine, which is a more hydrophobic residue in that position. It also had a histidine in the H2 position, which is bigger than a valine of SIP1 and has more propensity to pH changes that may alter the conformation of the ar/R selectivity filter under different conditions, suggesting a different function from the SIP1 subgroup. In this sense, in a yeast heterologous system, both SIP1s detected in *A*. *thaliana* (SIP1;1 and SIP1;2) demonstrated water-channel activity but not SIP2;1 [103].

Members of the XIP aquaporin subfamily have been functionally characterized as urea, glycerol, hydrogen peroxide, boric acid, copper, arsenic and nickel transporters, but impermeable to water [105]. However, mimicking the ar/R selectivity filter residues from AtTIP2;1 in *Nicotiana benthamiana* NbXIP1;1, allowed the permeability of water in such a channel [105]. The tridimensional model of NbXIP1;1 of that study suggests that, despite the pore size being wide enough for water permeability, the presence of hydrophobic residues may prevent the hydrogen bonding required for the passing of water molecules through the channel at the ar/R region. The PruavXIP1;1 also contained hydrophobic residues in the ar/R selectivity filter, but PruavXIP2;1 had a threonine in the H2 position, which could give the hydrogen bonding network required for water mobilization. In addition, the presence of a methionine at P1 of Froger’s residues in PruavXIP1;1 may have a structural function, stabilizing the protein chains, taking in account that this residue tends to form bonds with the amide groups of the nearest amino acids [106,107], suggesting different functions for both XIP aquaporins in *P. avium.*

### 4.2. Organ-Specific Expression

The presence of aquaporins has been widely documented in all plant organs or tissues, but the numerous isoforms present gene expressions subjected to temporal and spatial regulation [45,55,108,109,110]. Such transcriptional behavior may be associated with specific roles in the physiological processes of each organ. As expected, the analysis of transcript levels carried out by qPCR revealed a large mRNA variation of the different isoforms of *PruavAQPs* depending on the plant organ. In rice, expression of some root PIPs and root anatomical properties were correlated with hydraulic traits [111]. In addition, anatomical metanalysis data and qPCR analyses evidenced a root-associated expression of *OsNIP2*;*1*, *OsTIP2*;*1*, and *OsPIP2*;*3* in comparison with other *OsAQPs* [112]. In cherry, two *NIPs* (*PruavNIP5*;*1* and *PruavNIP5*;*2*), one *TIP* (*PruavTIP4*;*1*) and one *PIP* (*PruavPIP1*;*3*) were found to be preferentially expressed in root tissues.

At the leaf level, AQP transcriptional changes related to cell expansion and leaf elongation have been reported in Arabidopsis *AtTIP1*;*1* [113], maize *ZmPIP1*;*1*, *ZmPIP1*;*2*, *ZmPIP1*;*3*, *ZmPIP2*;*1* and *ZmPIP2*;*2* [114] and barley (*HvPIP1*;*1*, *HvPIP1*;*5*, *HvPIP2*;*2*, *HvPIP2*;*5*, *HvTIP1*;*1*, *HvTIP2*;*3* and *HvNIP1*;*1* [115]. Interestingly, *ZmAQP* transcriptional patterns were correlated with cell water permeability, suggesting an involvement of some AQPs in the leaf growth process. In our study, five *PruavAQPs* (*PruavNIP4*;*2, PruavTIP1*;*1, PruavTIP2*;*2, PruavTIP3*;*1 and PruavXIP1*;*1*) exhibited a remarkable mRNA accumulation. It should be noted that in this group there were no members of the PIP subfamily, some of which have CO_2_ transport capacity, but an XIP aquaporin was included as transcriptionally relevant in the context of this organ.

Flowering is the fundamental process that sustains the generation of offspring and the survival of seed plant species. Within the barley genome have been found four AQPs (*HvTIP1*;*1*, *HvTIP1*;*2*, *HvTIP2*;*3*, and *HvPIP2*;*1*), predominantly expressed in flowers, with *HvTIP1*;*1* having the highest expression among them [116]. In addition, in Arabidopsis, several members of the TIP subfamily (*AtTIP1*;*3*, *AtTIP2*;*1*, *AtTIP3*;*1* and *AtTIP5*;*1*) were specifically expressed in petals, pointing out their relevance in flowering [117]. Similarly, *RhTIP1*;*1* and *LoPIP1* and *LoPIP2* showed transcriptional profiles predominantly associated with petals in rose and lily, respectively [118,119].

Apparently, PIPs and TIPs would play an essential role in flower opening and petal expansion via water uptake. In cherry, *PruavPIP2*;*1*, *PruavPIP2*;*2*, *PruavPIP2*;*4*, *PruavNIP6*;*1* and *PruavNIP4*;*2* were grouped into a clade, defined by their marked gene expression in stems, being even higher at the flower level, which was more evident in the case of PIPs. Out of this clade, only two TIPs (*PruavTIP1*;*3* and *PruavTIP2*;*1*) showed relevant gene expression at the flower level. Our analysis showed two NIPs to be strongly expressed in flowers, which may be due to the fact that whole flower samples were used, and other reproductive tissues, other than petals, could require boron and/or H_2_O_2_ transport functions that justify the expression of *PruavNIP6*;*1* and *PruavNIP4*;*2*.

### 4.3. Expression Profiles of PruavAQPs under Abiotic Stresses

The water balance and the osmotic balance in plants can be negatively impacted as a consequence of abiotic stresses. Expectedly, the expression patterns of *Pruav AQPs* varied not only in different organs but also under different abiotic stresses.

Counterintuitively, root hypoxia, generally imposed by flooding or waterlogging, triggers a decrease in hydraulic conductivity, which impairs the availability of water in plant tissues and affects photosynthetic parameters [120]. Decreases in root hydraulic conductivity have been associated with PIP down-regulations [121]. On the other hand, the hypoxia-responsive NIP1;1 has been reported as a H_2_O_2_ transporter with a relevant role in detoxifying anaerobic-related molecules in cells [10,95]. In roots of cherry plants under hypoxia, *PruavXIP1*;*1*, *PruavXIP2*;*1* and *PruavNIP4*;*2* were strongly expressed. Instead, transcript levels showed only slight variations at the leaf level, with *PruavPIP2*;*1* and *PruavXIP2*;*1* being the only up-regulated genes, albeit to a modest extent. Usually, the transcriptional profiles of AQPs are comparatively opposite between root and leaf tissues, but *PruavXIP2*;*1* expression also was up-regulated in leaves of oxygen-deprived plants (Figure 6). This AQP seems to be an interesting candidate to investigate in order to further define its functionality in the whole plant and its utility as a molecular marker for phenotypic selection against hypoxia stress in plant breeding programs.

Restrictions in the water availability can reduce stomatal and root hydraulic conductance, altering the water homeostasis of the plant [122]. In this context, the ability of AQPs to mobilize water between cells and tissues is key in plant adaptation processes. In Arabidopsis, a considerable number of AQP genes were strongly down-regulated upon drought stress, i.e., *AtPIP1*;*3*, *AtPIP1*;*5*, *AtPIP2*;*2*, *AtPIP2*;*3*, *AtTIP1*;*1*, *AtTIP1*;*2*, *AtTIP2*;*1* and *AtTIP2*;*2*. However, after almost one day post re-hydration, their transcript levels returned to values like those detected for control plants. On the other hand, AtPIP1;4 and AtPIP2;5 were up-regulated under this stress [108]. Tobacco plants under drought stress reduced the expression of *NtPIP1*;*1* and *NtPIP2*;*1* to reduce osmotic hydraulic conductance in the roots [123]. In the same way, three *HvTIP* genes (*HvTIP1*;*1*, *HvTIP1*;*2* and *HvTIP2*;*3*) involved in water transport diminished their transcripts under severe drought, but the expression of another two (*HvTIP3*;*1* and *HvTIP4*;*1*) were induced. In addition, several genes returned to expression levels like those detected under optimal irrigation conditions [124].

Under drought treatment, PruavTIP4.1 was shown to be early and strongly down-regulated, but at the leaf level in stressed plants it did not exhibit any alteration. Along with this, *PruavNIP6*;*1*, *PruavNIP5*;*2*, *PruavNIP5*;*1* and *PruavTIP2*;*2* also showed a decrease in their transcripts, although to a greater extent later (72 h) (Figure 6). On the other hand, *PruavXIP2*;*1*, *PruavXIP1*;*1*, *PruavNIP4*;*2*, *PruavNIP2*;*1*, *PruavPIP2*;*1* and *PruavNIP4*;*1* were up-regulated in roots of water-restricted plants. Noteworthy, *PruavXIP2*;*1* and *PruavPIP2*;*1* gene expressions were induced also in leaves under drought stress (Figure 6).

Salinity lowers the osmotic potential of soil solutions, imposing water stress and nutritional imbalance in glycophytes [125]. To some extent, this water stress mimics the effects of drought so that common molecular and biological responses may be found between these two stresses. In a comparative study between two salt-stress-contrasting genotypes of barley, a reduction of hydraulic conductivity was associated with a low expression of *HvPIPs* after 24 h of 100 mM NaCl treatment [126]. Likewise, a reduction of transcripts of two PIPs (*BoPIP1* and *BoPIP2*) was reported for broccoli plants after an application of 80 mM NaCl [127]. Compared with drought, salt stress alters a greater number of genes in Arabidopsis [128] which may be due to the double nature of this stress, being both an osmotic stress, like that established in drought (acute phase), and an ionic stress (chronic phase). This transcriptional behavior was not evidenced in *P. avium* under salt stress. Here, most of them showed little or no variation with respect to the levels of transcripts detected in roots of control plants, with the exception of *PruavNIP4*;*2* and *PruavNIP7*;*1*, which showed remarkable gene repression and induction, respectively. In leaves, another member of the NIP subfamily (*PruavNIP6*;*1*) was repressed and a slight induction was found in *PruavPIP2*;*1* (Figure 6). Here, two members of the NIP subfamily, i.e., PruavNIP4;1 and PruavNIP6;1, could be interesting for developing molecular markers to assist the selection of *Prunus* rootstock progenies with better physiological performances under salt stress conditions.

Low temperatures can affect plants due to their inherent effects on cellular processes and due to a dehydration phenomenon caused by the freezing of water [129]. Moreover, after an acute drop, a prolonged cold stress can increase the root hydraulic conductivity, as reported in rice. Concomitantly, a coordinated up-regulation of several root AQP genes was observed, highlighting the transcriptional activation of *OsPIP2*;*5* [51]. In *A*. *thaliana*, cold acclimation induced the expression of *AtPIP1*;*4* and *AtPIP2*;*5*. Additionally, the increase in the amount of PIP2;5 protein was confirmed by immunoblotting. Single and double mutants of these AQPs showed greater sensitivity to freezing temperatures compared with wild-type plants [130]. GhTIP1;1, a vacuolar AQP with water channel activity, is normally expressed in roots and hypocotyl of cotton seedlings, but in response to low temperatures it is rapidly down-regulated in roots and strongly up-regulated in cotyledons. Yeast overexpressing GhTIP1;1 improved their survival to freezing temperatures [50]. In *P. avium* roots, the greatest transcriptional reconfiguration occurred at the earliest time of stress (6 h), where the marked up-regulation of *PruavNIP4*;*1*, *PruavXIP1*;*1*, *PruavXIP2*;*2*, *PruavTIP2*;*2* and *PruavNIP2*;*1* reached a peak of transcripts that then decreased at 72 h of cold stress, although in most of these cases exceeding the values detected in roots of control plants. It should be noted that *PruavNIP4*;*1* is the AQP most expressed in cherry roots subjected to cold temperatures, but it also shows this pattern in roots under high salinity, so it could be participating in a common response mechanism for both stresses. More studies are needed to understand the mechanisms involved in this fact. In leaves, the great and early accumulation of *PruavNIP2*;*1* transcript in response to cold is remarkable. Noteworthy, this stress is where this AQP shows its greatest transcriptional change, so it could become a selection marker to phenotype sensitivity to low temperatures in *Prunus* breeding.

High temperatures alter the structure of plant cells, causing an increase in membrane permeability and affecting osmoregulation. In addition, heat stress negatively impacts numerous metabolic and physiological processes and, depending on the severity of the stress, growth inhibition and even plant death [131,132,133,134]. A transcriptome analysis of *Rhazya stricta* leaves, an evergreen shrub native to extremely hot regions (Western and South Asia), showed the up-regulation of three AQP genes (*PIP2*;*1*, *PIP1*;*2* and *TIP2*;*1*) in response to high temperature, likely involved in thermotolerance in leaves and also in protection of the photosynthetic apparatus [135]. In *S*. *bicolor* (L.) exposed to heat stress, *SbPIP1*;*1*, *SbPIP1*;*2*, *SbTIP1*;*1*, *SbTIP3*;*1*, *SbNIP2*;*1* and *SbSIP1*;*2* were significantly up-regulated and *SbPIP1*;*5*, *SbPIP1*;*6*, *SbPIP2*;*1*, *SbPIP2*;*4*, *SbPIP2*;*6*, *SbTIP3*;*3*, *SbTIP4*;*2*, *SbTIP4*;*3* and *SbNIP3*;*2* were markedly repressed [73]. This last study showed a notable transcriptional reconfiguration at the AQPs level in this cereal species, which coincides with what was found in the case of cherry plant roots exposed to heat stress, where many members of *PruavPIPs*, *PruavNIPs*, *PruavTIPs* and *PruavXIPs* were up-regulated. However, in cherry leaves, only *PruavXIP2*;*1*, *PruavNIP4*;*2* and *PruavPIP2*;*1* increased their gene expression after 72 h of heat stress treatment. On the other hand, only *PruavTIP4*;*1* and *PruavTIP1*;*1* were strongly down-regulated in heat stressed roots and leaves, respectively. (Figure 6). It is interesting to note that in two related stresses, heat and drought, PruavNIP4;2 consistently showed an induction of its expression at 72 h of the respective treatments. More research is needed to understand the function of this AQP that emerges as a possible molecular marker to select *Prunus* progenies better adapted to semi-arid or arid environments where the combined stress of water restriction and high temperatures usually occurs.

## 5. Conclusions

Plant aquaporins have been demonstrated to be important membrane proteins controlling the flux of water and other solutes, all of which are key to the development and survival of plant organisms. There is a large and growing body of evidence showing roles of aquaporins in the plant response to stress, so these are proteins with an interesting capacity as targets for improving crop adaptation to adverse environmental conditions. In this work, we present a comprehensive study of *AQP* encoding genes in *P. avium* (‘Mazzard F12/1’ rootstock) on a genome-wide scale. A total of 28 non-redundant *AQP* genes were identified in *Prunus* spp. Genomes, which were phylogenetically grouped into five subfamilies (PIP, NIP, TIP, SIP and XIP). Bioinformatic analyses revealed a very high synteny and remarkable conservation of structural features among orthologs of different *Prunus* genomes. The differences detected at the level of gene promoters of the different *PruavAQPs* could account for the expression variations associated with each organ and abiotic stress (hypoxia, drought, salinity, cold and heat) analyzed in this study. Based on the transcriptional profiles at the root and leaf level of cherry plants subjected to abiotic stresses, possible candidate genes are proposed for further functional studies and for the development of molecular markers that can assist the progeny selection processes more efficiently in the context of breeding programs for rootstocks and/or varieties of stone fruit species better adapted to sustain food production, despite the challenges imposed by climate change.

## Figures and Tables

**Table 1:** Structural and subcellular localization analysis of *P. avium* aquaporins. SF: subfamily; AA: amino acids; WP: Wolf PSORT; PP: plant mPloc; TDP: hidden Markov model transmembrane domain prediction; Mw: molecular weight (kDa); Ip: isoelectric point; FR: Froger’s residues; Ka/Ks: nonsynonymous (Ka) and synonymous (Ks) calculation. GRAVY: grand average of hydropathy. Plas: plasma membrane; Vacu: vacuole; CM: cell membrane; VC: vacuole.

SF	AQP	AA	NPA I	NPA II	ar/R	WP	PP	TDP	Mw	Ip	FR	Ka/Ks	GRAVY
	PruavNIP1;1	281	NPA	NPA	WVAR	Plas	CM	6	29.77	9.30	FSAYI	0.6	0.432
	PruavNIP2;1	291	NPA	NPA	GSGR	Plas	CM	6	30.91	8.64	LTAYV	0.63	0.287
	PruavNIP4;1	267	NPA	NPA	WVAR	Plas	CM	6	28.18	6.41	LSAYF	0.17	0.722
NIP	PruavNIP4:2	248	NPA	NPA	WVAR	Plas	CM	6	26.06	6.40	LSAYI	0.19	0.732
	PruavNIP5;1	298	NPS	NPI	SIGR	Plas	CM	6	31.26	7.64	FTAYI	0.11	0.517
	PruavNIP5;2	280	NPS	NPV	A-GR	Vacu	CM	5	29.28	8.85	FTAYL	0.58	0.298
	PruavNIP6;1	307	NPS	NPV	TIAR	Plas	CM	6	31.89	8.34	FTAYL	0.14	0.415
	PruavNIP7;1	300	NPA	NPA	AVGR	Plas	CM	6	31.99	5.82	YSAYI	0.23	0.535
	PruavPIP1;1	286	NPA	NPA	FHTR	Plas	CM	5	30.79	9.28	ESAFW	0.45	0.282
	PruavPIP1;2	290	NPA	NPA	FIGR	Plas	CM	6	30.85	9.20	QSAFW	0.36	0.379
	PruavPIP1;3	286	NPA	NPA	FHTR	Plas	CM	6	30.66	9.25	QSAFW	0.31	0.331
PIP	PruavPIP2;1	287	NPA	NPA	FHTR	Plas	CM	6	30.52	8.94	QSAFW	0.25	0.511
	PruavPIP2;2	281	NPA	NPA	FHTR	Plas	CM	6	30.04	7.00	QSAFW	0.38	0.420
	PruavPIP2;3	284	NPA	NPA	FHTR	Plas	CM	6	30.07	6.89	QSAFW	0.29	0.505
	PruavPIP2;4	281	NPA	NPA	FHTR	Plas	CM	6	29.83	9.00	QSAFW	0.41	0.415
	PruavSIP1;1	240	NPS	NPA	AVPN	Vacu	CM.VC	5	25.18	9.82	MAAYW	0.35	0.896
SIP	PruavSIP1;2	244	NPT	NPA	TVPN	Chlo	CM.VC	6	25.88	9.52	MAAYW	0.22	0.755
	PruavSIP2;1	236	NPL	NPA	SHGS	Plas	CM	4	25.80	9.52	FVAYW	0.53	0.538
	PruavTIP1;1	252	NPA	NPA	HIAV	Plas	VC	6	25.94	5.54	TSAYW	0.18	0.769
	PruavTIP1;2	252	NPA	NPA	HIAV	Vacu	VC	6	26.08	4.78	TSAYW	0.25	0.884
	PruavTIP1;3	252	NPA	NPA	HIAV	Plas	VC	6	26.15	6.12	TSAYW	0.41	0.735
TIP	PruavTIP2;1	248	NPA	NPA	HIGR	Plas	VC	7	25.40	6.26	TSAYW	0.55	0.941
	PruavTIP2;2	248	NPA	NPA	HIGR	Plas	VC	6	25.17	4.86	TSAYW	0.44	0.982
	PruavTIP3;1	256	NPA	NPA	HIGR	Plas	VC	6	27.26	7.11	TAAYW	0.58	0.530
	PruavTIP4;1	249	NPA	NPA	HIAR	Vacu	VC	7	26.24	5.54	TSAYW	0.54	0.821
	PruavTIP5;1	255	NPA	NPA	NVGC	Plas	CM.VC	6	25.84	6.81	VAAYW	0.06	0.842
XIP	PruavXIP1;1	304	SLV	SPA	VIVR	Plas	CM	6	32.17	5.97	MCAFW	0.43	0.725
	PruavXIP2;1	314	NPV	NPA	ITVR	Plas	CM	7	33.85	7.74	VCAFW	0.55	0.665

**Table 2:** Functional inference based on the specificity-determining positions analysis of sweet cherry aquaporins. Differences in residues are marked with grey.

Substrate	Aquaporin	Specificity-Determining Positions								
		SDP1	SDP2	SDP3	SDP4	SDP5	SDP6	SDP7	SDP8	SDP9
Ammonia Transporter		F/T	K/L/N/V	F/T	V/L/T	A	D/S	A/H/L	E/P/S	A/R/T
	PruavTIP2;1	T	L	I	L	A	T	H	P	V
Boric Acid Transporter		T/V	I/V	H/I	P	E	I/L	I/L/T	A/T	A/G/K/P
	PruavPIP1:1	T	I	H	P	E	L	L	T	P
	PruavPIP1:2	T	I	H	P	E	L	L	T	P
	PruavPIP1:3	T	I	H	P	E	L	L	T	P
	PruavPIP2;1	T	I	H	P	E	I	L	T	P
	PruavPIP2;2	T	I	H	P	E	I	L	T	P
	PruavPIP2;3	T	I	H	P	E	I	L	T	P
	PruavPIP2;4	T	I	H	P	E	I	L	T	P
	PruavNIP1;1	V	I	H	P	E	L	M	A	P
	PruavNIP2;1	V	I	H	P	E	I	I	A	P
	PruavNIP4;1	V	I	H	P	E	I	F	A	P
	PruavNIP4;2	V	I	H	P	E	L	F	A	P
	PruavNIP5;1	V	I	H	P	E	V	L	A	P
	PruavNIP5;2	V	I	H	P	E	L	L	A	P
	PruavNIP6;1	V	I	H	P	E	L	L	A	E
	PruavNIP7;1	I	I	H	P	E	L	L	T	P
	PruavXIP2;1	T	I	H	P	E	I	T	T	V
CO_2_ Transporter		I/L/V	I	C	A	I/V	D	W	D	W
	PruavPIP1;1	V	I	T	A	I	D	W	D	W
	PruavPIP1;3	V	I	T	A	I	D	W	D	W
	PruavPIP2;2	V	I	S	A	V	D	W	D	W
H_2_O_2_ Transporter		A/S	A/G	L/V	A/F/L/T/V	I/L/V	H/I/L/Q	F/Y	A/V	P
	PruavNIP2;1	S	A	L	L	V	I	Y	V	P
	PruavNIP4;1	S	A	L	L	V	L	Y	A	P
	PruavNIP5;1	S	A	L	V	I	L	Y	V	P
	PruavPIP1;1	A	G	V	F	I	H	F	V	P
	PruavPIP1;2	A	G	V	F	I	H	F	V	P
	PruavPIP1;3	A	G	V	F	I	H	F	V	P
	PruavPIP2;1	A	G	V	F	I	Q	F	V	P
	PruavPIP2;2	A	G	V	F	I	Q	F	L	P
	PruavPIP2;3	A	G	V	F	I	Q	F	V	P
	PruavPIP2;4	A	G	V	I	I	Q	F	V	P
	PruavTIP1;1	S	A	L	A	I	H	Y	A	P
	PruavTIP1;3	A	A	L	V	I	H	Y	V	P
	PruavTIP2;1	A	A	L	V	I	N	Y	V	P
	PruavTIP2;2	S	A	L	V	I	N	Y	V	P
	PruavTIP3;1	A	A	L	V	I	H	Y	V	P
	PruavTIP5;1	S	A	L	T	I	Q	Y	V	P
	PruavXIP2;1	A	G	L	V	V	H	F	V	P
Silicic acid Transporter		C/S	F/Y	A/E/L	H/R/Y	G	K/N/T	R	E/S/T	A/K/P/T
	PruavNIP2;1	S	F	V	H	G	N	R	S	N
Urea Transporter		H	P	F/I/L/T	A/C/F/L	L/M	A/G/P	G/S	G/S	N
	PruavNIP1;1	H	P	I	A	L	P	G	S	N
	PruavNIP2;1	H	P	L	A	M	P	G	S	N
	PruavNIP5;1	H	P	I	A	L	P	G	S	N
	PruavNIP5;2	H	P	I	A	L	P	G	S	N
	PruavPIP1;1	H	P	F	F	L	P	G	G	N
	PruavPIP1;2	H	P	F	F	L	P	G	G	N
	PruavPIP1;3	H	P	F	F	L	P	G	G	N
	PruavPIP2;1	H	P	F	F	L	P	G	G	N
	PruavPIP2;2	H	P	F	F	L	P	G	G	N
	PruavPIP2;3	H	P	F	F	L	P	G	G	N
	PruavPIP2;4	H	P	F	F	L	P	G	G	N
	PruavTIP1;1	H	P	F	F	L	A	G	S	N
	PruavTIP1;2	H	P	F	F	L	A	G	S	N
	PruavTIP1;3	H	P	F	A	L	P	G	S	N
	PruavTIP2;1	H	P	F	A	L	P	G	S	N
	PruavTIP2;2	H	P	L	A	L	P	G	S	N
	PruavTIP3;1	H	P	F	L	L	P	G	S	N
	PruavTIP4;1	H	P	L	L	L	A	G	S	N
	PruavTIP5;1	H	P	F	A	L	P	G	S	N
	PruavXIP1;1	H	L	F	A	V	G	G	G	N

## Data Availability

Data are available on request from the corresponding author.

## Supplementary Materials

The following supporting information can be downloaded at: https://www.mdpi.com/article/10.3390/genes14040940/s1, Table S1: List of qPCR primers for *PruavAQPs*.
