# Supplementary material for: Genome-Wide Identification and Gene Expression Analysis of Sweet Cherry Aquaporins (Prunus avium L.) under Abiotic Stresses
Source: Genes (Basel). 2023 Apr 19;14(4):940. doi: 10.3390/genes14040940 (PMC10138167; doi:10.3390/genes14040940)
Supplement: Supplementary file 1 [file genes-14-00940-s001.zip › genes-2350353-supplementary.pdf]

Table S1: List of qPCR primers for *PruavAQPs*.

Gene	Forward Primer (5' - 3')	Reverse Primer (5' - 3')
PruavPIP1;1	AGGGAAAGAAGAAGATGTGAGG	GTGATGTAGAGGAACAAGAAGGTG
PruavPIP1;2	CCTGGTTTCCTTTCGTTTTTA	AATGGTCGGTGTTATTTCTGA
PruavPIP1;3	GCTTGGGATGACCATTGGATC	AGGAAAATTAGCCCCTGGACTTG
PruavPIP2;1	GATATAGTCAGGTTGAAAAGGGA	GAAGAAAGGAGGAGAGATGGA
PruavPIP2;2	ATTCTGAGAGCAGGAGCCATTA	CTTTGAGCCCTTGAGAGAGACA
PruavPIP2;3	GGGTTTGCTGTGTTTATTGTG	GCTCCTGCTCTCAGTATGTATTG
PruavPIP2;4	CTTTGGTCCTGCTGTGATTTTC	CTTCCTTTTTCATCTCCCTCTCT
PruavNIP1;1	CAAACCCAACCAAGAATCAAGCAG	ACCATCACAACCAGTCCCCAAA
PruavNIP2;1	AGCACATACTACAAGGGAATCTGG	CAAATGAGCGTGGTGGAGGT
PruavNIP4;1	CGTGGTATTTATCGGGTGTGG	GCGAAAGATGGCAAAGGTG
PruavNIP4;2	CCAGGAACCAATACAAGCGA	CACAGCCACCGAACCACAC
PruavNIP5;1	GCAGGACCTATTGTGAATGAGAA	TATTATGTAGAGGGGACTTGGG
PruavNIP5;2	AAGTGCTTGCCTGTGAAGGC	ATGAGGATGAAGGTTCCGACAA
PruavNIP6;1	CAGAAGCAGTGGGATTGGTAG	AAGTGAGACAGGAGGGTGAGG
PruavNIP7;1	ATCAAGAAATGGGCTCTAATGC	ATAAAAGTCCCCACCATCTCTG
PruavTIP1;1	GGTCACATTACCGTCTTCCG	AGTATCCCACCCCACTTGC
PruavTIP1;2	CTGGTGGTGCGTTTGATGGT	TTGTTGGGTAGGGGTTCGTG
PruavTIP1;3	ACTGACGGTGCTTCAACGAC	CAAGACAGACCTGACGAGAGAGAT
PruavTIP1;4	AACATTTTGGCGGGTGGGG	AGTAATCAGCGGTCGGGAGTGG
PruavTIP2;1	GCACCCTTAGTCAACGAGTATT	GCCTGGCTTCACCAACTTC
PruavTIP2;2	AGGGTTCACTGGGAATCATAGCA	AATGAAAATGTCACCATAGACGAGC
PruavTIP3;1	GATAGCGGCACATCAGCAGC	GCCACAATAGCCCCAAGGAG
PruavTIP5;1	CTGTTGTGGGCTCTATGATGTC	CCATCTGAGAAATCCAATAGCA
PruavSIP1;1	TTTCACAGTCATCATCGCCTCG	GCAGTGGTGGAGGGGTTGAA
PruavSIP1;2	ACTCAAGGGTCCTCGTAGCC	CTATGAAAGGGCAAATCCAAT
PruavSIP2;1	CTGTGATGGGATGGGCTTATG	TCTACCTTTTCCTCTTTTATGTGTGG
PruavXIP1;1	GTGGGCATAACAGTGGCTTT	GCCGTCCCTGTGATTGTTAT
PruavXIP2;1	ACCAATGGGGCTAGGAGTTT	CTTACCAATGCAGGGCCTAA
